# Complication after knee intra-articular betamethasone injection, a thrombocytopenic purpura: a case report

**DOI:** 10.1186/s13256-026-05873-8

**Published:** 2026-03-31

**Authors:** Marine Fauny, Audrey Fresse, Damien Loeuille, Isabelle Chary-Valckenaere

**Affiliations:** 1 Department of Rheumatology, University Hospital, Nancy, France; 2 Department of Rheumatology, Saint Charles Hospital, Toul, France; 3 Regional Center of Pharmacovigilance, Pharmacology-Toxicology Department, University Hospital, Nancy, France

**Keywords:** Purpura, Thrombocytopenic, Intra-articular injection, Betamethasone, Adverse drug reaction, Pharmacovigilance

## Abstract

**Background:**

Intra-articular corticosteroid injections represent a therapeutic option for the management of various pain conditions; however, they are not devoid of the potential for adverse drug reactions. In this study, we present a rare systemic adverse drug reaction that has not been previously described: thrombocytopenic purpura.

**Case presentation:**

A 73-year-old white female patient suffering from severe acute knee osteoarthritis was administered an intra-articular corticosteroid injection (betamethasone) for the second time. Within a few hours, she developed generalized skin lesions characterized by purpuric lesions all over her skin. The mucosae were excluded from this phenomenon. A thrombocytopenia was associated. A skin biopsy revealed the presence of purpura, accompanied by leukocytoclastic vasculitis and immune deposits of the immunoglobulin A type. A thorough medical examination was conducted, including a comprehensive allergy assessment yielding negative results. However, a positive irritative intradermo-reaction for betamethasone was observed. The skin lesions regressed spontaneously within a period of 2 weeks.

**Conclusion:**

The corticosteroid injection was likely to have caused the symptoms owing to the following evidence: firstly, the acute clinical presentation (only a few hours after the injection); secondly, the rapid improvement in the patient’s condition; and thirdly, the absence of further signs indicating other serious complications. Thrombocytopenic purpura, a rare systemic adverse drug reaction, has not been previously described.

## Background

Intra-articular corticosteroid injections represent a therapeutic option for the management of various pain conditions (for example, osteoarthritis); however, they are not without the potential for adverse drug reactions (ADRs).

Some complications after intra-articular corticosteroid injections are well documented [1–8], such as septic arthritis, skin depigmentation or atrophy, hyperpigmentation, diabetes or hypertension imbalance, and microcrystal reaction. In the relevant literature, other clinical presentations include facial flushing and the presence of both Nicolau and Tachon syndromes have been described. A number of cases of genuine allergies, accompanied in some cases by anaphylactic reactions, have also been reported. However, the occurrence of purpuric skin lesions was not usually described after corticosteroid injection.

## Case report

A 73-years-old white female patient suffering from knee osteoarthritis presented with acute pain, swelling, and functional impairment in her right knee. Radiographic imaging was used to confirm the presence of osteoarthritis, with a Kellgren and Lawrence grade of 3 being assigned to the external femorotibial joint line.

Two years prior, intra-articular steroid injection (betamethasone) and acid hyaluronic injection allowed improvements in pain and functional performance.

Clinically, there was a swollen knee, with movement limitation. A decision was made to proceed with a puncture, in which 36 mL of citrine-yellow liquid were removed, in addition to an intra-articular corticosteroid injection of betamethasone to improve pain and the functional limitation. The patient presented a vagal discomfort after the injection, which spontaneously resolved within minutes. The following day, the patient attended the rheumatology department with skin lesions. Following the injection, the patient reported experiencing a general pruritus and purpuric erythematous skin lesions on the trunk and limbs, accompanied by headaches and arthralgia. Antihistaminic medications have been demonstrated to offer an improvement of pruritus. Subsequently, symptoms such as diarrhea, abdominal pain, and asthenia were observed.

The patient’s medical history did not include any recent instances of allergic reactions, infections, or the introduction of new treatments.

**Fig. 1:**
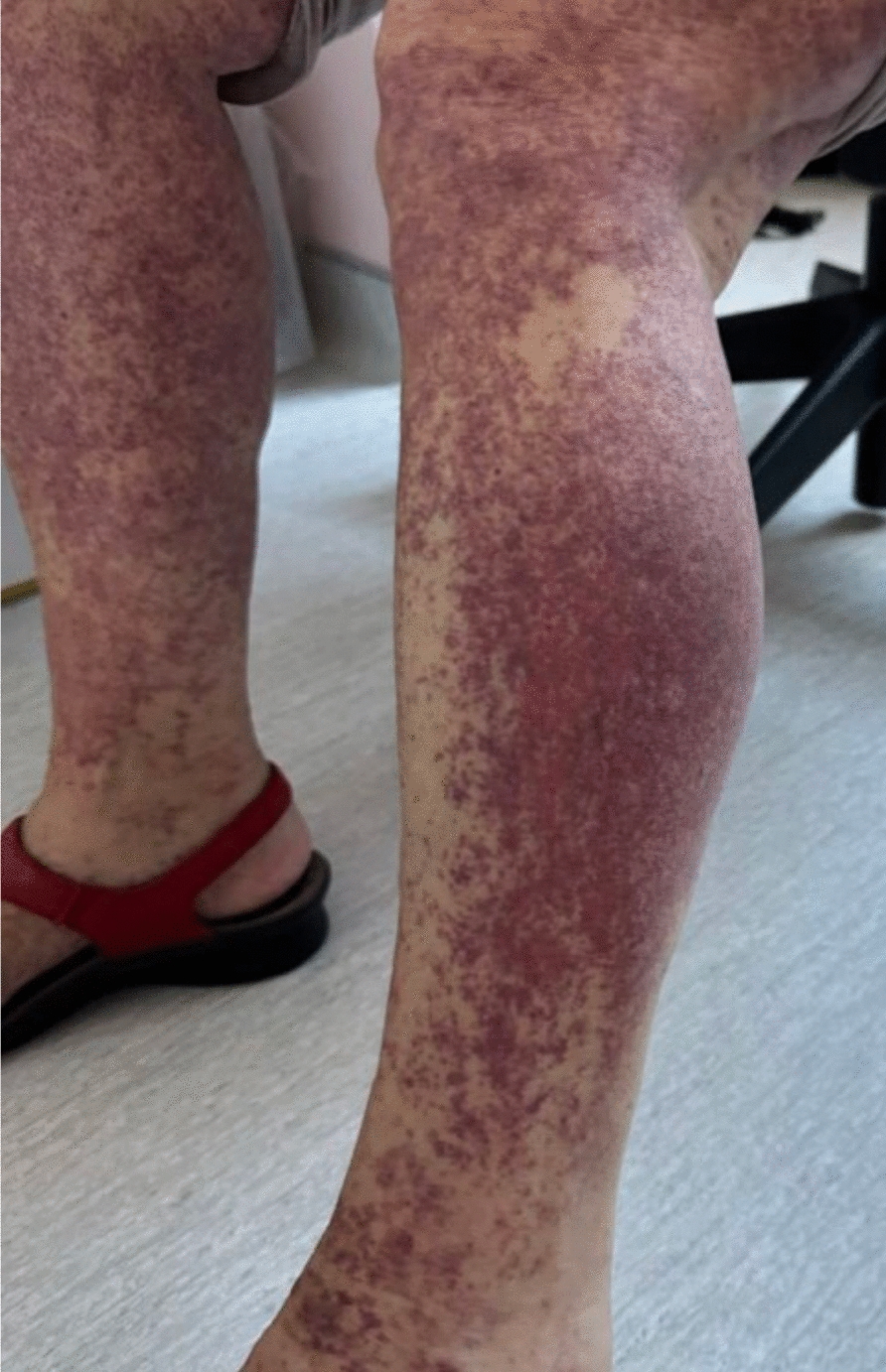
Purpuric skin lesions after knee intra-articular betamethasone injection

A thrombocytopenia was identified (95 G/L versus 239 G/L 2 months prior). A clinical examination of the limbs and trunk revealed the presence of purpuric skin lesions, with no evidence of mucosal involvement (Fig. 1).

Two days later, there was an improvement in thrombocytopenia (124 G/L), neutrophilia (15.8 G/L), low CH50, C3, and C4 without autoimmune antibody (anti-citrullinated protein antibodies (ACPA), rheumatoid factor, extractable nuclear antigen (ENA), antinuclear, and Anti-neutrophil cytoplasmic antibody (ANCA)), serologies (HIV, hepatitis B and C, syphilis) were also negatives. A skin biopsy revealed the presence of purpura, accompanied by leukocytoclastic vasculitis and immune deposits of the immunoglobulin A (IgA) type.

A thorough medical examination was conducted, including a comprehensive allergy assessment yielding negative results. However, a mild irritant reaction to betamethasone was observed.

The skin lesions regressed over a period of 2 weeks, along with the other symptoms.

In fact, this complication could be either rheumatoid purpura or immune-allergic vasculitis. This examination does not permit the retention of a single diagnosis. To avoid recurrence of these symptoms, betamethasone is now contraindicated and monitoring of biology and proteinuria is being performed.

## Discussion

In rare instances, the administration of local corticosteroids can result in unintended consequences, including ADRs. These reactions may manifest as a range of symptoms, such as allergy, erythematous rash, Tachon and Nicolau syndromes, anaphylactic shock, palpitations, and occasionally, psychiatric disorders. The occurrence of thrombocytopenic purpura had not been previously documented.

A summary of betamethasone product characteristics reports purpura (without specifications) only for systemic use.

In literature, a single case of local purpura has been documented following a period of 5 years of betamethasone (topical use) [9].

The WHO international pharmacovigilance database, VigiBase, and the French Pharmacovigilance Database (FPVD) were queried on 10 January 2024 for all reported cases with at least one suspected and/or interacting drug containing betamethasone and at least one adverse drug reaction corresponding to MedDRA classification high level term (HLT) “purpura related conditions.” Cases were included in our analysis if they included at least one ADR related to thrombocytopenia and/or if they mentioned thrombocytopenia in the case’s narrative.

Similar cases occurring after betamethasone local injection/infiltration, but without thrombocytopenia, were also analyzed in a separate part.

VigiBase and FPVD extractions recorded 88 and 40 cases of betamethasonse induced-purpura, respectively, from which we identified 5 cases of thrombocytopenic purpura after betamethasone use (1 Italian and 4 French). The characteristics of the cases are presented in Table 1. Five cases of non-thrombocytopenic purpura cases after betamethasone local injection/infiltration were also identified in the FPVD.

**Table 1:** Case characteristics with at least one suspected and/or interacting drug containing betamethasone and at least one adverse drug reaction corresponding to MedDRA classification high level term (HLT) “purpura related conditions”

	Patient (gender and age)	Serious case	Betamethasone	Other suspected drugs	ADR	TTO	Outcome
Thrombocytopenic cases							
Case 0 (our case)	♀ 73 Y.O.	Yes: hospitalization	Single intramuscular injection	None	Thrombocytopenic purpura. Platelets: 95 G/L	2 hours	Not recovered
Case 1 (IT)	♂ 65 Y.O.	Yes: hospitalization	1.5 mg per day, intramuscular form	Amoxicillin/clavulanic acid, ceftriaxone	Thrombocytopenic purpura with epistaxis and hematuria	1 day	Favorable after treatment stop
Case 2 (FR)	♂ 22 Y.O.	Yes: hospitalization	2 mg per day, oral form	Clarithroymicin, tixocortol/bacitracine	Thrombocytopenic purpura with gingivorrhagia. Platelets: 2G/L	13 days	Favorable after treatment stop, corticotherapy and platelets transfusion
Case 3 (FR)	♀ 86 Y.O.	Yes: hospitalization	Cutaneous application	Spironolactone/altizide, hydroxyzine, rilménidine	Thrombocytopenic purpura with gingivorrhagia. Platelets: 4 G/L	1 day	Favorable after treatment stop and corticotherapy
Case 4 (FR)	♀ 85 Y.O.	Yes: hospitalization	1 application per day, cutaneous form	Fluindione, pristinamycin,	Purpura associated with generalized erythema and increased INR. Platelets: 98 G/L	29 days	Favorable
Non-thrombocytopenic cases							
Case 5 (FR)	♀ 47 Y.O.	No	Single intramuscular injection	None	Edematous purpura in patches on the external surfaces of the limbs	1 day	Favorable
Case 6 (FR)	♂ 49 Y.O.	No	Single intramuscular injection	None	Purpura of lower body associated with hepatic cytolysis, asthenia, and weight loss	1 day	Favorable
Case 7 (FR)	♀ 48 Y.O.	Yes: hospitalization	Single intra-articular infiltration	Meglumine ioxaglate, lidocaine	Purpura associated with arthromyalgia and syncope	6 hours	Favorable
Case 8 (FR)	♂ 36 Y.O.	No	Single intra-articular infiltration	D-penicillamin, esomeprazole, isradipine	Ecchymosis of lower body. Normal platelet count	2 days	Favorable
Case 9 (FR)	♀ 20 Y.O.	No	Single intramuscular injection	None	Purpura associated with hot flushes on face and neck	1 day	Favorable

*ADR* adverse drug reaction, *FR* French, *INR* International Normalized Ratio, *IT* Italian, *TTO* time to onset, *Y.O.* years old

WHO international pharmacovigilance database VigiBase and French Pharmacovigilance Database (FPVD) were queried on 10 January 2024 for all reported cases with at least one suspected and/or interacting drug containing betamethasone and at least one adverse drug reaction corresponding to MedDRA classification high level term (HLT) “purpura related conditions”

## Conclusion

It has been demonstrated that the development of thrombocytopenic purpura may be a complication that arises subsequent to the administration of an intra-articular corticosteroid injection. In such cases, it is not possible to determine whether rheumatoid purpura or immune-allergic vasculitis is responsible for the observed reaction.

It is imperative that physicians possess the ability to provide reassurance to patients. It is important to note that no specific treatment is required. It is imperative that a systematic and continuous monitoring process be implemented for blood platelets and symptoms, with the objective of ensuring the spontaneous resolution of the condition. The medication involved should be avoided in order to prevent the reappearance of these symptoms.

## Data Availability

Not applicable.
